# Sanqi Oral Solution Ameliorates Renal Ischemia/Reperfusion Injury via Reducing Apoptosis and Enhancing Autophagy: Involvement of ERK/mTOR Pathways

**DOI:** 10.3389/fphar.2020.537147

**Published:** 2020-09-16

**Authors:** Ruimin Tian, Pinchao Wang, Lihua Huang, Chuang Li, Zhaoyu Lu, Zhisheng Lu, Aijun Wu, Kun Bao, Wei Mao, Qingming Huang, Peng Xu

**Affiliations:** ^1^ State Key Laboratory of Dampness Syndrome of Chinese Medicine, The Second Affiliated Hospital of Guangzhou University of Chinese Medicine, Guangzhou, China; ^2^ The Second Clinical Medical College, Guangzhou University of Chinese Medicine, Guangzhou, China; ^3^ Department of Nephrology, Guangdong Provincial Hospital of Chinese Medicine, Guangzhou, China; ^4^ Guangdong Provincial Academy of Chinese Medical Sciences, Guangzhou, China; ^5^ Guangdong Provincial Key Laboratory of Chinese Medicine for Prevention and Treatment of Refractory Chronic Diseases, Guangzhou, China; ^6^ Department of Pediatrics, Guangdong Provincial Hospital of Chinese Medicine, Guangzhou, China

**Keywords:** renal ischemia-reperfusion (I/R), acute kidney injury (AKI), Radix Astragali, Radix Notoginseng, apoptosis, autophagy, extracellular signal-regulated kinase (ERK), mammalian target of rapamycin (mTOR)

## Abstract

Ischemia-reperfusion (I/R) induced acute kidney injury (AKI) is a significant health problem with high morbidity and mortality, yet prophylaxis strategies and effective drugs are limited. Sanqi oral solution (SQ) is a formulated medicine widely used in clinical settings to treat various renal diseases via enriching qi and activating blood circulation while its role on I/R-AKI remains unclear. Herein, by establishing rat I/R-AKI models, we intended to investigate the effect of SQ on the prevention of I/R-AKI and explore its underlying mechanisms. We demonstrated that SQ treatment significantly attenuated renal dysfunction of I/R-AKI, alleviated histological damages, inhibited renal apoptosis, and enhanced autophagy. Further investigation proved that SQ could significantly inhibit the activation of ERK and mTOR signaling pathways. Moreover, its renoprotective effect can be abolished by autophagy inhibitor 3-methyladenine (3-MA). Collectively, our results suggest that SQ exerts renoprotective effects on renal I/R injury via reducing apoptosis and enhancing autophagy, which are associated with regulating ERK/mTOR pathways.

## Introduction

Acute kidney injury (AKI) is a worldwide health problem associated with high morbidity, mortality, and heavy socioeconomic burdens (Chertow et al., 2005; Bellomo et al., 2012; Lewington et al., 2013; Zuk and Bonventre, 2016). Besides acute consequences, AKI can easily deteriorate and further lead to chronic kidney disease or even end-stage renal disease (Venkatachalam et al., 2010; Leung et al., 2013). Renal ischemia/reperfusion (I/R) injury is usually caused by hypovolemic shock, kidney transplantation, cardiac or vascular surgery, trauma, and heart infarction (Jakobsson et al., 2014; Benck et al., 2015), and therefore serves as one of the leading causes of AKI in clinical settings (Malek and Nematbakhsh, 2015; Zuk and Bonventre, 2016). However, the current treatment options of I/R-AKI still largely remain supportive management, and prophylaxis strategies and effective drugs are indeed limited.

Chinese herbal medicine has been adopted to effectively treat various diseases for thousands of years based on the essence of “treatment based on syndrome differentiation”. From the perspective of traditional Chinese medicine (TCM), the pathogenesis of I/R-AKI can be classified as Qi deficiency and blood stasis, which may be prevented or cured through the replenishment of Qi and activation of blood circulation. Sanqi oral solution (SQ), consisting of Radix Astragali (*Astragalus membranaceus Fisch. ex Bunge*) and Radix Notoginseng [*Panax notoginseng (Burkill) F.H.Chen*], is a patented formula and ready-made pharmaceutical preparation in Guangdong Provincial Hospital of Chinese Medicine (Wei et al., 2013; Tian et al., 2019; Xu et al., 2019). It is an effective formulated product to treat some renal diseases via benefiting Qi and activating blood, yet its role on I/R-AKI remains unclear.

Increasing investigations have substantiated the possible pathological mechanisms underlying I/R-AKI over the past decades, nevertheless, the complex pathophysiology of I/R-AKI has not been fully understood. It is a complicated process which involves initial injury in the early stage of ischemia and secondary leision after reperfusion. During acute ischemia, the renal blood flow is restricted which leads to the primary injury of renal cells. After reperfusion, blood and oxygen are reintroduced into renal tissues, which aggravate the injury by cascade reactions including oxidative stress, inflammation increase, cellular apoptosis, autophagy, necrosis, or even ferroptosis (Sutton and Molitoris, 1998; Sheridan and Bonventre, 2000). Among the abovementioned research advances, apoptosis and autophagy are two critical possible mechanisms in the pathogenesis of I/R-AKI. Apoptosis is a form of cell suicide mediated by caspase activation and is essential for development and homeostasis. Accumulating reports have indicated that I/R-induced cell death is closely associated with apoptosis (Oberbauer et al., 2001; Bonegio and Lieberthal, 2002; Yingjie et al., 2019). Autophagy is a process of delivering long-lived proteins, cellular macromolecules, and intracellular organelles to lysosomal for degradation and recycling. By recycling toxic material into new cellular components, autophagy supports anti-stress responses and energy maintenance in response to numerous stresses (Levine and Kroemer, 2008; Mizushima and Komatsu, 2011). According to published records, autophagy may be either up-regulated or down-regulated in I/R-AKI models, and act as a "double-edged sword" which may ameliorate or exacerbate renal injury (Baehrecke, 2005; Suzuki et al., 2008; Jiang et al., 2010; Decuypere et al., 2014; Decuypere et al., 2015).

In our previous study, we have identified that the major compositions of SQ include ginsenosides, notoginsenoside R1, calycosin, and so on (Tian et al., 2019; Xu et al., 2019). Among these compositions, ginsenoside Rg 1, notoginsenoside R1 and calycosin are capable of reducing apoptosis (Chen et al., 2001; Liu B. et al., 2016; Huang et al., 2017; Tong et al., 2019; Zhai et al., 2019), notoginsenoside R1 and calycosin also exhibit effects on activating autophagy in HT29 cells, podocytes or renal mesangial cells (Huang et al., 2017; Wen et al., 2020). Yet, their roles on renal I/R injury and tubular cells have never been elucidated and deserves to be addressed.

Given these findings, we intended to investigate whether SQ can ameliorate renal injury in I/R-AKI rats and explore the mechanisms involved, focusing mainly on apoptosis and autophagy. Our results demonstrate that SQ exerts renoprotective effects on renal I/R injury via reducing apoptosis and enhancing autophagy, which are associated with regulating ERK/mTOR pathways.

## Materials and Methods

### Chemicals and Reagents

Pierce® BCA Protein Assay Kit was obtained from Thermo Scientific. Bax (2772), cleaved Caspase 3 (9664), SQSTM1/p62 (5114), Beclin1 (3495), p-ERK1/2 (4370), ERK1/2 (4695), p-mTOR (5536), mTOR (2983), HRP-linked anti-rabbit IgG (7074), HRP-linked anti-mouse IgG (7076) antibodies, and DAB Substrate Kit were purchased from Cell Signaling Technology. Bcl-2 (ab196495) antibody was obtained from Abcam. LC3 (M186-3) was purchased from MBL International. β-actin (BM0627) was from Boster Biological Technology. Enhanced chemiluminescence (ECL) reagent was obtained from Millipore. In Situ Cell Death Detection Kit (11684795910) was obtained from Roche Diagnostics GmbH. 3-Methyladenine (3-MA) were purchased from Selleck Chemicals.

### Preparation and Analysis of SQ

SQ was mass manufactured by Guangdong Provincial Hospital of Chinese Medicine (Guangzhou, China) and approved by the Drug Administration of Guangdong Province to produce for clinical use (Cantonese medicine production number: Z20071155). In line with the standards of Chinese Pharmacopoeia, SQ was extracted from two crude drugs: Radix Astragali and Radix Notoginseng, at a concentration of 0.333 and 0.056 g/mL, respectively, whose botanical name of each herb can be checked and validated in https://mpns.science.kew.org/mpns-portal/. Their botanical samples (batch number: 181111) have been kept for posterity in the Pharmaceutical Preparation Department of Guangdong Provincial Hospital of Chinese Medicine and can be accessed from in the future whenever needed.

Furthermore, in order to ensure the quality of SQ in the present study, the major chemical components of SQ were profiled. In brief, the analysis for quality control was carried out by an Agilent 1200 HPLC system with DAD detector. The LC separation was performed over a Kinetex C18 column (4.6 × 100 mm, 2.6 μm, Phenomenex Inc., Torrance, USA) at 30 °C. Samples were eluted by gradients according to the elution program as following: 0–12 min, 88–80% A; 12–26 min, 80–74% A; 26–40 min, 74–35% A. The UV detection wavelengths were set at 205 and 284 nm.

Since SQ has been chemically characterized and batch produced by Guangdong Provincial Hospital of Chinese Medicine, it holds the same composition and chemical profiles as described in our previous article (Xu et al., 2019). The composition and chemical constituent analysis are shown in **Supplementary Table S1** and **Supplementary Figure S1**.

According to the clinical guidelines of SQ, its human-rat equivalent dosage was calculated. For rats in the current study, 6.3 mL/kg/d (low dose) or 12.6 mL/kg/d (high dose) of SQ was administered intragastrically.

### Animals

Adult male Sprague-Dawley rats weighting 200–250 g were provided by the Medical Experimental Animal Center of Guangdong Province and maintained in Experimental Animal Center of Guangdong Provincial Hospital of Chinese Medicine. The rats were housed under standard environmental conditions (21–25 °C, 50-60 % humidity, and 12 h/12 h light/dark cycle) and allowed free access to water and food. All of the procedures were performed in accordance with the international guidelines for the care and use of laboratory animals and approved by the Institutional Animal Care and Use Committee at Guangdong Provincial Hospital of Chinese Medicine.

### Renal I/R Experiment

All rats were randomly divided into four groups: (1) sham group, (2) I/R-AKI model group, (3) I/R-AKI + SQ-low group, and (4) I/R-AKI + SQ-high group. The I/R-AKI model rats were anesthetized with intra-peritoneal injection of 1% pentobarbital sodium (40 mg/kg), and received renal ischemia, with bilateral pedicles clamped by nontraumatic clamps for 40 min, and then 24 h reperfusion. Successful ischemia and reperfusion were confirmed visually by the immediate change in the kidney color (ischemia: from red to atropurpureus, and reperfusion: from atropurpureus to red). The sham control rats were subjected to the identical surgery except for renal pedicle clamping. For other two groups, SQ was administrated daily by oral gavage at the dose of 6.3 mL/kg/d (SQ-low) or 12.6 mL/kg/d (SQ-high) from 7 days before the operation to the next day after the surgery, and the rats underwent the same surgical procedures as I/R-AKI model group. A corresponding volume of distilled water was administered to rats in sham and model groups. In this experiment, the success rate of modeling was 91%.

Within 24 h, there were 2 rats died, one of which in the model group, and another one in I/R-AKI + SQ-low group, leading to a 6.25% mortality rate. After 24 hours’ reperfusion, all rats were sacrificed and blood samples were collected. Kidneys were removed and preserved for further analysis.

### Renal Function Detection

In this study, we measured blood urea nitrogen (BUN) and serum creatinine (SCR) levels to detect the renal function. Blood samples were collected and then centrifuged in condition of 3,000 rpm for 15 min at room temperature. The serum samples were analyzed by the clinical laboratory of Guangdong Provincial Hospital of Chinese medicine.

### Histological Analysis

All rats were anesthetized with pentobarbital sodium and sacrificed. Kidneys were fixed with 4% buffered paraformaldehyde for 48 h and embedded in paraffin. The paraffin were then sectioned into 3 μm thick and stained with hematoxylin and eosin (H&E) to estimate the tissue damage.

### Immunohistochemistry

Immunohistochemical assay was used to the measure the expression of cleaved Caspase 3 in kidney slices. The kidneys were fixed, paraffin-embedded and cut into 3 μm slices. After being dewaxed and hydrated, the tissue slices were sequentially incubated with 3% hydrogen peroxide (H_2_O_2_) to block endogenous peroxidase activity, with albumin bovine fraction for blocking, and with anti-cleaved Caspase 3 antibody (1:500) overnight. Followed by secondary antibody and 3,3’3′-Diaminobenzidine (DAB) kit, the samples were examined under Olympus IX71 microscope (bright-field) equipped with an Olympus DP73 digital camera. Ten fields were randomly selected for each sample. The brown color was considered as positive staining according to the standard protocol.

### TUNEL Assay

Terminal transferase dUTP nick end labeling (TUNEL) staining was employed to identify apoptotic cells in renal tissues. The tissue slices were dewaxed and rehydrated according to the standard protocol, then were incubated with proteinase K working solution (10–20 μg/mL in 10 mM Tris/Hcl, pH 7.4–8) for 30 min at room temperature. After rinsing twice with PBS, each slice was added 50 μL TUNEL reaction mixture which was obtained by adding 5 μL enzyme solution to 450 μL label solution. The slides were incubated in a humidified atmosphere for 60 min at 37°C in the dark. After rinsing with PBS for 3 times, samples were embedded with antifade and analyzed under a fluorescence microscopy. For quantification, 10 representative fields were selected from each tissue section and the amount of TUNEL positive cells per mm^2^ was evaluated.

### Western Blot Analysis

The renal tissues were weighted to extract protein and their concentrations were detected by Pierce® BCA Protein Assay Kit. The protein samples (20–50 μg) were then resuspended in 5× sample buffer and boiled, and electrophoresed on a 10 or 15% sodium dodecyl sulfate-polyacrylamide gel electrophoresis (SDS-PAGE) and transferred onto PVDF membranes. Membranes were incubated with blocking buffer for 2 h, hybridized with primary antibodies (SQSTM1/p62, Beclin1, LC3, Bcl-2, Bax, cleaved Caspase 3, p-Erk1/2, Erk1/2, p-mTOR, mTOR, 1:1,000) overnight at 4 °C and followed by secondary antibodies (HRP-labeled anti-rabbit or anti-mouse IgG, 1:3,000, 2 h). The protein bands were visualized using ECL reagent, then signals were captured and quantified by Image Lab System (Bio-Rad Laboratories, Inc.).

### Statistical Analysis

All experiments were performed at least three times independently. Statistical analysis of all data was performed by using SPSS 22.0 and GraphPad Prism 7. The data were presented as means ± standard deviation (SD). Statistical comparisons were carried out by one-way analysis of variance (ANOVA) followed by Tukey’s test. *p<0.05 was considered to indicate a statistically significant difference.

## Results

### Identification of Chemical Constituents in SQ

SQ was identified by HPLC/MS and the chemical constituents are shown in **Supplementary Figure S1**.

### SQ Attenuates I/R-Induced Renal Dysfunction and Histopathological Changes

**Figure 1:**
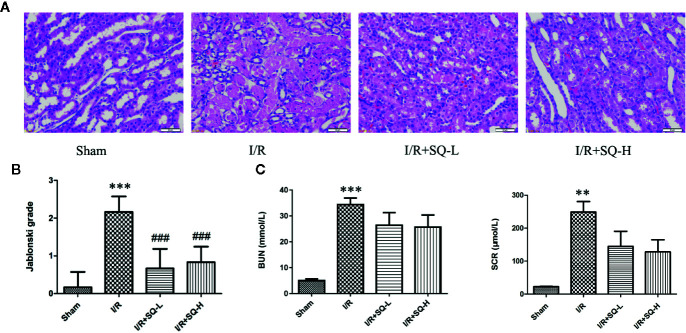
SQ attenuates I/R-induced renal dysfunction and histopathological changes. **(A)** Representative HE staining of renal tissues. **(B)** Jablonski grade to reflect tubular injury calculated from HE staining. **(C)** BUN and SCR levels which indicated the function of kidney filtration. Data were presented as means ± SD, **p < 0.01 and ***p < 0.001 versus Sham group, ^###^p < 0.001 versus I/R group. n = 6 in each group.

Serum creatinine (SCR) and blood urea nitrogen (BUN) are two biological indexes to indicate the function of kidney filtration (Baum et al., 1975; Finco and Duncan, 1976). AKI is characterized by an abrupt decrease of the glomerular filtration rate, and thus leading to creatinine and urea retention in plasma (Bellomo et al., 2012; Zuk and Bonventre, 2016; Manoeuvrier et al., 2017), so we firstly detected the SCR and BUN levels in all groups. As expected, compared with sham group, the I/R model exhibited dramatic higher levels of SCR and BUN. Treatment of SQ partly antagonized these changes, with partly reduction of SCR and BUN (**Figure 1**).

Histological examination by H&E staining can reveal the lesions in kidney,and Jablonski grade is the standard histopathological method to estimate the injury of kidney tubules (Jablonski et al., 1983). Therefore, we next conducted histological analysis. From the H&E staining, we can clearly observe that the renal I/R injury led to obvious cell swelling, intraluminal necrotic cellular debris, interstitial congestion, and luminal narrowing in renal tissues. Significantly, SQ treatment ameliorated these damages. Accordingly, Jablonski grade was notably increased in I/R-AKI group in comparison with the sham group. In contrast to the model group, SQ significantly reduced the histopathologic grade (**Figure 1**).

### SQ Reduces Apoptosis in I/R-AKI Rats

**Figure 2:**
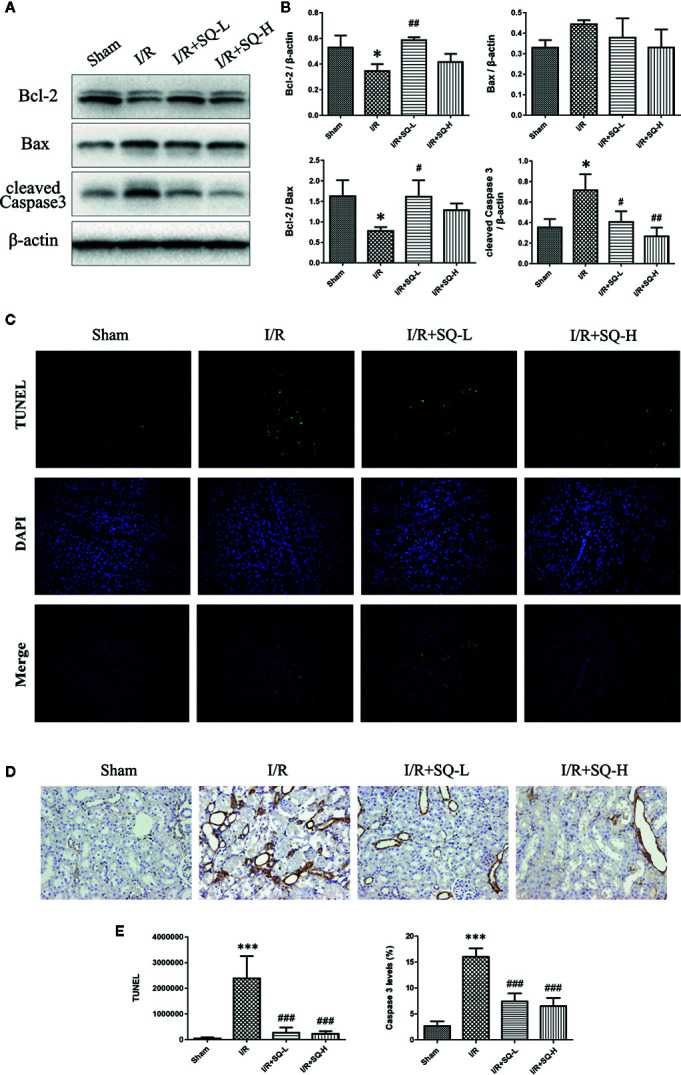
SQ reduces apoptosis in I/R-AKI. **(A)** Representative western blot of apoptosis-related proteins: Bcl-2, Bax, and cleaved Caspase 3. **(B)** Quantitative analysis of western blot results. β-actin was used as loading control. **(C)** Representative images of TUNEL assay in each group. **(D)** Representative images of immunohistochemical assay of cleaved Caspase 3 expression in each group. **(E)** Quantitative analysis of TUNEL and immunohistochemical stainings. Data were presented as means ± SD, *p < 0.05 and ***p < 0.001, versus Sham group, ^#^p < 0.05, ^##^p < 0.01, and ^###^p < 0.001 versus I/R group. n = 3 in each group.

Apoptosis is a programmed cell death characterized by energy-dependent biochemical mechanisms and morphologic changes, including shrinkage of the cell and nucleus, chromatin condensation, and DNA fragmentation, followed by rapid engulfment of the cellular corpse by macrophages and neighboring viable epithelial cells (Bonegio and Lieberthal, 2002). Ample evidences have corroborated that apoptosis are involved in renal tubular epithelial cells in I/R-AKI (Kaushal et al., 2004; Havasi and Borkan, 2011; Yang et al., 2019; Yingjie et al., 2019), thus the extent of apoptosis was assessed in renal tissues by TUNEL staining, which labels 3’-OH ends of DNA by endonucleases that are activated during apoptosis. Our results showed that I/R injury led to the number of TUNEL-positive cells dramatically increase in renal tissues, while SQ blocked the changes significantly. To better understand the molecular mechanism, we performed Western blot to test the changes of apoptosis-related proteins. As shown in **Figure 2**, up-regulation of pro-apoptotic protein Bax and down-regulation of anti-apoptotic protein Bcl-2 were observed in the I/R model group, which were reversed by SQ treatment. Furthermore, apoptosis marker cleaved Caspase 3, was tested by Western blot and then confirmed by immunohistochemical assay. Western blot bands showed that I/R injury strikingly enhanced the levels of cleaved Caspase 3 in renal tissues, which can be counteracted by SQ treatment, in a dose-dependent manner. In line with the immunoblotting, immunohistochemistry results of cleaved Caspase 3 revealed similar trends.

### SQ Enhances Autophagy in I/R-AKI Rats

**Figure 3:**
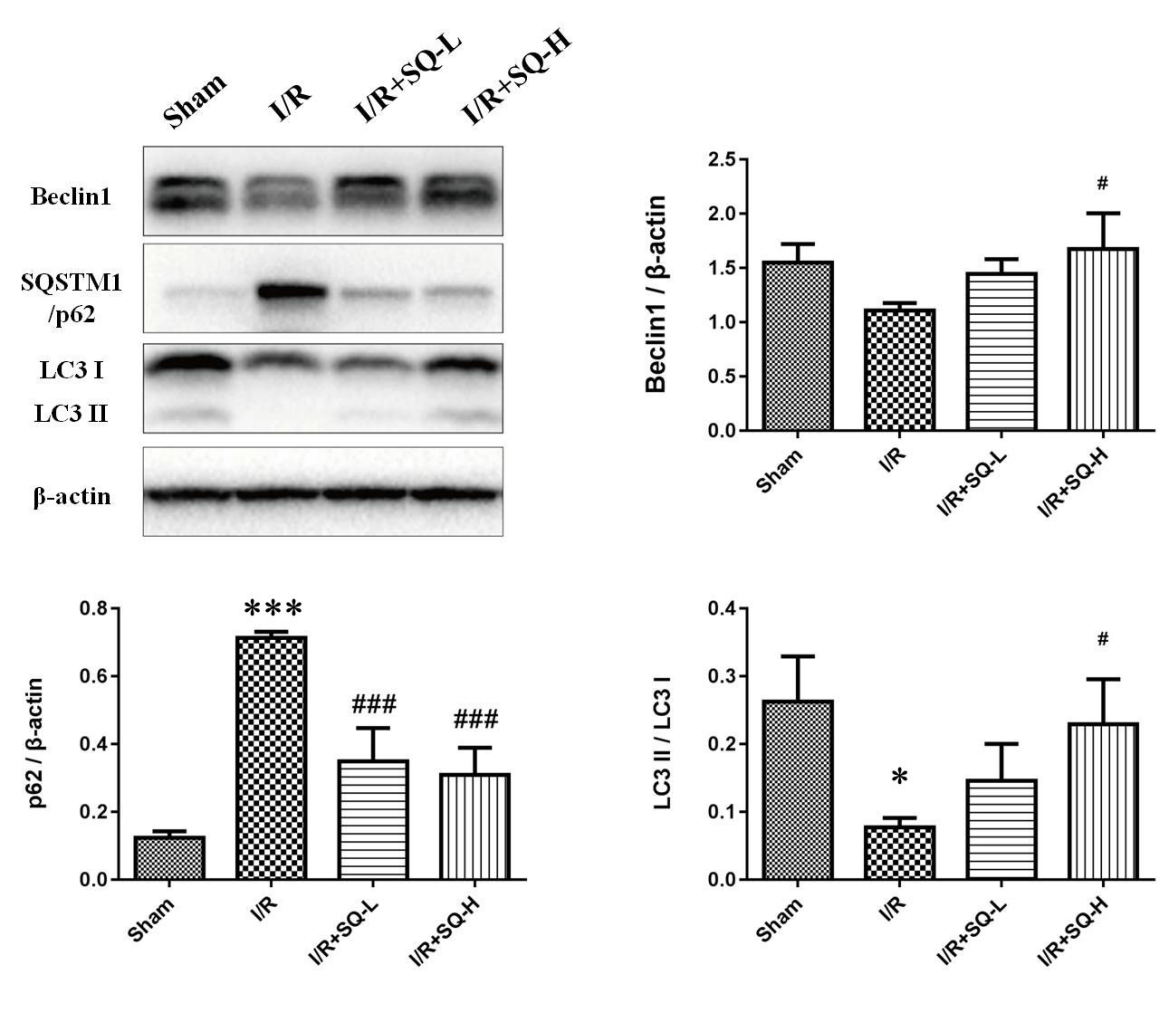
SQ enhances autophagy in IR-AKI. Representative western blot of autophagy markers: Beclin1, SQSTM1/p62, and LC3, and the corresponding quantitative analysis. β-actin was used as loading control. Data were presented as means ± standard deviation (SD), *p < 0.05 and ***p < 0.001 versus Sham group, ^#^p < 0.05 and ^###^p < 0.001 versus I/R group. n = 3 in each group.

In order to determine whether autophagy was implicated in the process of I/R-AKI and drug treatment, we tested autophagy-related proteins by Western blot. To our surprise, compared with the sham-operated controls, the model group displayed down-regulated autophagy, with decrease in LC3II/LC3I, striking increase of SQSTM1/p62 and mild elevation of Beclin1. In contrast to the model group, higher dose of SQ enhanced autophagy, with significant increase of LC3II/LC3I and Beclin1, as well as marked decline of SQSTM1/p62 (**Figure 3**).

### SQ Protects Renal I/R Injury Through Regulating ERK/mTOR Pathways

**Figure 4:**
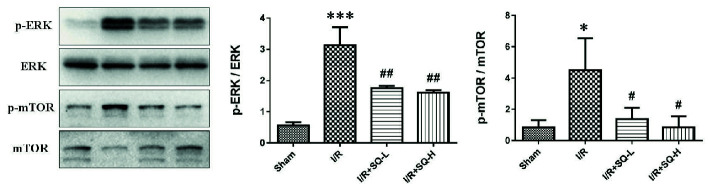
Effects of SQ on ERK and mTOR signaling pathways in I/R-AKI. Representative western blot of p-ERK, ERK, p-mTOR, and mTOR, and the corresponding quantitative analysis. Data were presented as means ± SD, *p < 0.05 and ***p < 0.001 versus Sham group, ^#^p < 0.05 and ^##^p < 0.01 versus I/R group. n = 3 in each group.

It has been reported that extracellular signal-regulated kinase 1/2 (ERK1/2) is abnormally activated during apoptosis (Sabbatini et al., 2006; Zeng et al., 2012; Liu D. et al., 2016; Kong et al., 2019), thus closely related to the pathological process of I/R injury. Given that SQ suppressed I/R-induced oxidative stress and apoptosis, we sought to explore the possible changes of ERK1/2 activation. Western blot analysis revealed that renal I/R injury induced a distinct activation of ERK, as a result of ERK1/2 phosphorylation, which could be recovered by SQ treatment (**Figure 4**). Moreover, considering that the pathway of mammalian target of rapamycin (mTOR) have been shown to inhibit autophagy in various models and cell types (Kim and Guan, 2015), we also checked whether SQ had an influence on mTOR. Western blot bands showed that the level of p-mTOR/mTOR was significantly increased upon I/R injury, which could be diminished by SQ treantment, in a dose-dependent manner (**Figure 4**). Taken together, our results suggested that ERK and mTOR pathways are involved in the protective effect of SQ.

### The Protective Effect of SQ Could Be Blocked by Autophagy Inhibitor 3-MA

Since the above data demonstrated that SQ was capable of reducing apoptosis and enhancing autophagy, these findings also prompted us to investigate the possible relationship of apoptosis and autophagy during the process. For this purpose, autophagy inhibitor 3-MA was utilized. Although the low and high doses of SQ exerted indistinguishable effects of attenuating I/R-induced renal dysfunction and histopathological changes (without statistically difference), the high dose showed better capability of reducing apoptosis (**Figure 2**) as well as enhancing autophagy (**Figure 3**). Therefore, we applied the high dose of SQ in later experiment. Rats were randomly divided into four groups: sham control, I/R-AKI model, I/R-AKI + SQ-high, and I/R-AKI + SQ-high + 3-MA. The operation was induced using the same method described before (Methods: renal I/R experiment), with a 92% success rate of modeling in this round of experiment. SQ was administrated daily by oral gavage at the dose of 12.6 mL/kg/d from 7 days before the operation to the next day after the surgery. 3-MA was intraperitoneally injected at the dose of 400 nM before the wounds of belly were sutured during the surgery.

Within 24 h, there were 2 rats died, one of which in the model group, and another one in I/R-AKI + SQ-high + 3MA group, leading to a 6.25% mortality rate. After 24 hours’ reperfusion, rats were sacrificed and blood samples were collected. Kidneys were removed and processed for analysis.

By performing HE staining and renal function detection, we found that SQ combined with 3-MA showed worse histological damages and renal functions than SQ alone (**Figure 6**). Consistently, further analysis by Western blot, immunohistochemistry and TUNEL assays revealed that, in comparison with SQ alone, 3-MA combination treatment aggravated renal damages by increasing apoptosis (**Figure 7**).

## Discussion

AKI is a worldwide health problem with high morbidity and characterized by rapid deterioration of kidney function (Chertow et al., 2005; Bellomo et al., 2012; Lewington et al., 2013; Zuk and Bonventre, 2016). Renal I/R injury occurs in lots of clinical settings, such as hypovolemic shock, kidney transplantation, cardiac or vascular surgery, trauma, heart infarction, and therefore serves as one of the leading risk factors for perioperative AKI (Malek and Nematbakhsh, 2015). Despite the improvement in understanding and management of I/R-AKI in recent years, prophylaxis strategies and effective drugs are still limited up till now. As a formulated medicine, SQ have been utilized in clinic to treat renal diseases for more than 20 years, mainly through benefiting Qi, promoting blood circulation, and removing stasis. Moreover, some of the components in SQ have been scientifically proven to possess anti-apoptosis or autophagy-regulating properties. Previous researches have reported that Astragalus membranaceus plus Panax notoginseng can synergistically ameliorate renal injury in diabetic nephropathy partly through inhibiting apoptosis (Zhai et al., 2019) or promoting autophagy (Wen et al., 2020). Calycosin, one of the major components in Astragalus membranaceus, has been verified to inhibit oxidative stress-induced cardiomyocyte apoptosis via activating estrogen receptor-α/β (Liu B. et al., 2016). Another active component Astragaloside IV can not only suppress apoptosis in mammary epithelial cells (Wang F. et al., 2019) or cerebral I/R Injury (Zhang et al., 2019), but also enhance autophagy via mTORC1 signalling (Lin et al., 2020) or SIRT-NF-κB p65 axis (Wang X. et al., 2019), and can reduce complement membranous attack complex induced podocyte injury through decreasing ERK expression (Zheng et al., 2012). Ginsenoside Rg1, the main component in Panax notoginseng, was capable of protecting pheochromocytoma (PC12) cells against dopamine-induced apoptosis (Chen et al., 2001). Meanwhile, notoginsenoside R1 has been documented to play therapeutic effects on various organs I/R Injuries (Guo et al., 2019; Tong et al., 2019), as well as attenuate glucose-induced podocyte injury via the inhibition of apoptosis and the activation of autophagy through PI3K/Akt/mTOR pathway (Huang et al., 2017). Nevertheless, the role of SQ on renal I/R injury have never been elucidated. Therefore, in the current study, by establishing rat I/R-AKI model, the renoprotective effect of SQ and its underlying mechanisms were demonstrated.

AKI is defined as sudden reduction or even loss of kidney function resulting in the accumulation of end products of nitrogen metabolism (urea) and creatinine (Bellomo et al., 2012; Zuk and Bonventre, 2016; Manoeuvrier et al., 2017), so we firstly detected the SCR and BUN levels in all groups. Herein, following a standard protocol, we clamped both renal pedicles for 40 min followed by 24 h recovery to establish the I/R-AKI model. BUN, SCR, and histopathological scores were detected after I/R injury, which can be used to evaluate the level of tubular damage and renal function. The experimental evidence showed that SQ can partly attenuate renal dysfunction and significantly decrease Jablonski grades in I/R-AKI rats (**Figure 1**), which presents the effects on protecting renal injury and tubular damage.

In the I/R-AKI, both in ischemia and reperfusion stages, there exist the imbalance of local tissue oxygen supply and demand as well as accumulation of waste products of metabolism. As a consequence of this mismatch, the tubular epithelial cells undergo injury and with increasing time or severity of injury, leading to various cell death pathways including necrosis, apoptosis, autophagy, or ferroptosis, which codetermine the cell fate of death or survival, thus resulting in organ functional impairment of water and electrolyte homeostasis and reduced excretion of waste products of metabolism (Bonventre and Yang, 2011). For decades, apoptosis has been proposed as one of the leading cell death pathway responsible for the pathogenesis of I/R-AKI (Bonegio and Lieberthal, 2002; Havasi and Borkan, 2011). TUNEL staining well labeled 3’-OH ends of DNA by endonucleases that are activated during apoptosis and therefore gave us the direct manifestation of apoptosis. Caspases are a family of structurally related cysteine proteases that play a key role in the execution of apoptosis. Bax and Bcl-2, two members in Bcl-2 family, are regulatory factors of extreme importance in apoptosis. Bax is a proapoptotic protein which enhances apoptosis by causing mitochondrial membrane depolarization, while Bcl-2 is an antiapoptotic protein which inhibits apoptosis by preventing cytochrome C release into the cytoplasm (Kaushal et al., 2004). In line with previous researches, our results also showed that I/R injury induced distinct apoptosis in renal tissues, evidencing by the increase of TUNEL-positive cells, up-regulation of cleaved Caspase 3 and Bax, as well as down-regulation of Bcl-2. Furthermore, SQ treatment reversed above changes, suggesting its antiapoptotic effects (**Figure 2**).

In recent years, with the increasing insight into mechanisms, there is a growing consensus that apoptosis and autophagy are intricately connected in various diseases including I/R-AKI (Chien et al., 2007; Kaushal, 2012; Suzuki et al., 2019; Zhao et al., 2019). Autophagy is a highly regulated process of delivering long-lived proteins, cellular macromolecules, and intracellular organelles to lysosomal for degradation and recycle, which supports anti-stress responses and energy maintenance (Levine and Kroemer, 2008; Mizushima and Komatsu, 2011). The microtubule-associated protein light chain 3 (LC3) takes a central part in the formation of autophagic vacuoles and detection of LC3 I to LC3II conversion is the most reliable method to assess the level of autophagy (Mizushima and Yoshimori, 2007; Klionsky et al., 2016). SQSTM1/p62 can bind LC3, thus serving as a selective substrate of autophagy. Inhibition of autophagy often correlates with increased level of p62, and decreased p62 level is associated with autophagy activation (Larsen et al., 2010). Beclin1, the mammalian homologue of the yeast Agt6, forms a protein complex with class III phosphatidyl inositol-3 kinase within the autophagosome and may indicate the level of autophagy in some extent (Cao and Klionsky, 2007). Autophagy has been proved to be closely implicated in the process of I/R-AKI, but it may be either up-regulated or down-regulated, and have dual roles in different situations (Decuypere et al., 2015). The concept of a dual effect of autophagy has been supported by the findings of all the reports on in vivo autophagy modulation in renal I/R injury: autophagy was protective when ischemia time was short (20–40 min), but detrimental after more prolonged ischemia (40–60 min) (Decuypere et al., 2014). Ischemia of 40 min likely represents the ‘‘fatal’’ turning point above which autophagy transforms from a cell survival into a cell death pathway in the examined models, but the turning point will likely vary according to the species, strain, gender and the type (warm or cold) and length of ischemia in the studied model (Decuypere et al., 2014). Moreover, prolonged ischemia in I/R-AKI is often associated with a decrease of autophagic flux rather than an increase for the following reasons: the consumption and depletion of essential autophagic components after long starvation periods, the reactivation of mTOR activity and the inhibition of master regulators of autophagy such as ATG3, AMBRA1 and Beclin1 by caspases, which are activated in response to prolonged stress (Pallet, 2014).

In our study, by detecting the expression level of LC3, Beclin1 and SQSTM1/p62, we found that autophagy was decreased in I/R-AKI rats when ischemia for 40 min and reperfusion for 24 h, which matched the results of I/R-AKI models in which applied the same ischemia period (40 min) (Nakagawa et al., 2012; Lempiainen et al., 2013; Decuypere et al., 2015). However, the reason why autophagy varies in different ischemia period remains unclear and dynamic autophagy changes need to be explored in future work. Contrasting to I/R-AKI model rats, higher dose of SQ could significantly enhance autophagy, with the significant increase of LC3II/LC3I and Beclin1, as well as the marked decline of SQSTM1/p62 (**Figure 3**).

To better explore the protective mechanism of SQ in I/R-AKI, we further examined some pathway-related proteins. ERK signaling has been documented to be abnormally activated during apoptosis (Collier et al., 2016; Liu D. et al., 2016; Kong et al., 2019), and mTOR pathway plays a vital role in mediating autophagy (Kim and Guan, 2015). Western blot bands revealed that renal I/R injury increased the phosphorylation of ERK and mTOR, which could be counteracted by SQ treatment, in a dose-dependent manner (**Figure 4**).

Although the crosstalk between ERK and mTOR signaling pathway has not been specifically addressed in previous studies, several lines of evidence suggest that they may be interacted in some conditions (Krab et al., 2008; Liu et al., 2019). ERK may influence TSC1/2 to regulate mTOR (Ma et al., 2005; Zhu et al., 2014), or collaborated with mTOR via phosphatase DUSP6 (Bermudez et al., 2008). Our findings demonstrated their crosstalk in renal I/R injury and SQ treatment. In agreements with numerous investigations which have proved that mTOR pathway negatively regulate autophagy, herein, the variation trends of mTOR in different groups (**Figure 4**) were consistent with the changes of autophagy markers (LC3, Beclin1 and SQSTM1/p62) in **Figure 3**.

**Figure 5:**
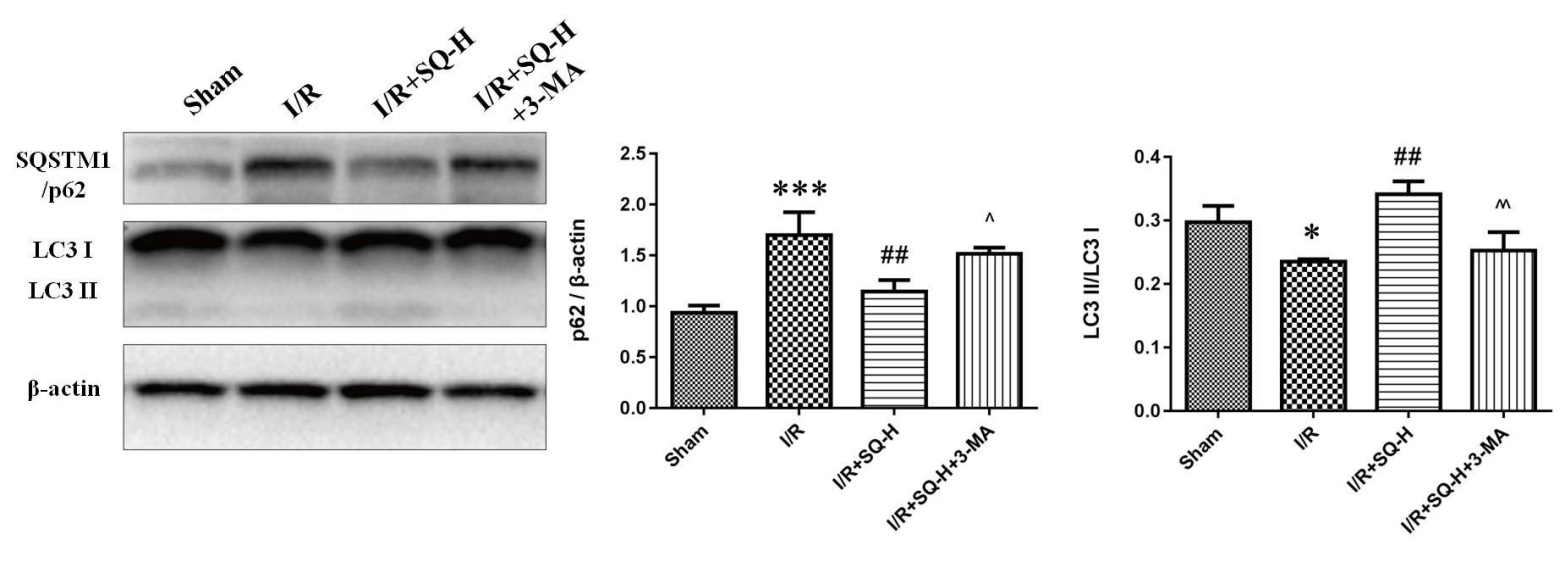
3-MA suppresses autophagy in I/R-AKI rats with SQ treatment. Representative western blot of PI3K III related autophagy protein SQSTM1/p62 and LC3, and the corresponding quantitative analyses. β-actin was used as loading control. Data were presented as means ± SD, *p < 0.05 and ***p < 0.001 versus Sham group, ^##^p < 0.01 versus I/R group, ^p < 0.05, ^^p < 0.01 versus I/R+SQ-H group. n = 3 in each group.

**Figure 6:**
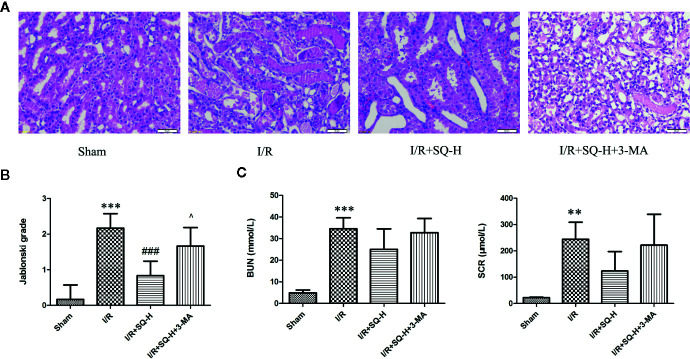
3-MA aggravates renal damages in I/R-AKI rats with SQ treatment. **(A)** Representative HE staining of kidney tissues. **(B)** Jablonski grade to reflect tubular injury calculated from HE staining. **(C)** BUN and SCR to indicate the function of kidney filtration. Data were presented as means ± SD, **p < 0.01, and ***p < 0.001 versus Sham group, ^###^p < 0.001 versus I/R group, ^p < 0.05 versus I/R+SQ-H group. n = 6 in each group.

**Figure 7:**
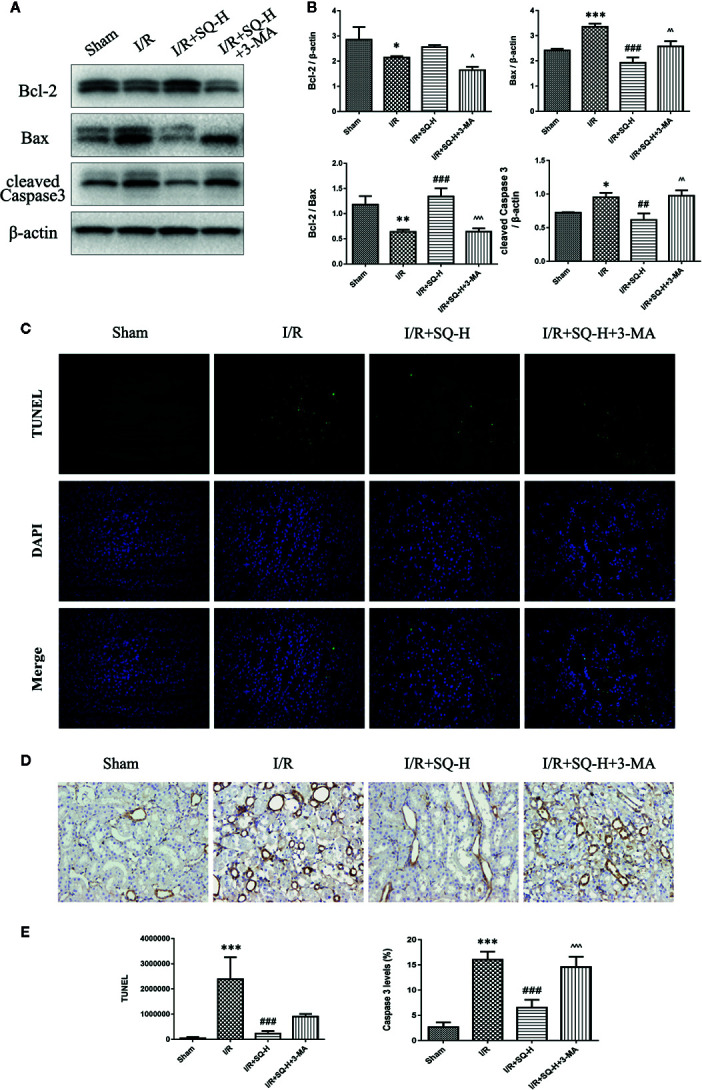
3-MA enhances apoptosis in I/R-AKI rats with SQ treatment. **(A)** Representative western blot of apoptosis-related protein Bcl-2, Bax, Caspase 3 **(B)** Quantitative analysis of western blot results. β-actin was used as loading control. **(C)** Representative images of TUNEL assay in each group. **(D)** Representative images of immunohistochemical assay of cleaved Caspase 3 expression in each group. **(E)** Quantitative analysis of TUNEL and immunohistochemical stainings. Data were presented as means ± SD, *p < 0.05, **p < 0.01, and ***p < 0.001 versus Sham group, ^##^p < 0.01, and ^###^p < 0.001 versus I/R group. ^p < 0.05, ^^p < 0.01, ^^^p < 0.001 versus I/R+SQ-H group. n = 3 in each group.

Given the findings that SQ reduced apoptosis and enhanced autophagy in I/R-AKI, we next intended to investigate the possible relationship of apoptosis and autophagy during the process. 3-Methyladenine (3-MA) is a PI3K inhibitor that inhibits autophagosomes sequestration at an early stage resulting in reduced LC3-II/LC3-I and the number of autophagy structures (Seglen and Gordon, 1982). To further confirm the renoprotective effect of autophagy and SQ, 3-MA was administered in I/R-AKI + SQ rats. Compared with I/R-AKI + SQ group, the ratio of LC3-II/LC3-I significantly decreased while the expression of p62 significantly increased in the I/R-AKI + SQ + 3-MA group, indicating that 3-MA inhibited autophagy successfully (**Figure 5**). By performing HE staining and renal function detection, we can clearly observe that inhibiting autophagy (I/R-AKI + SQ + 3-MA) aggravated histological damages and renal function (**Figure 6**). Later analysis by Western blot, immunohistochemistry and TUNEL assays, revealed that inhibiting autophagy led to the exacerbation of apoptosis during the process (**Figure 7**).

## Conclusion and Future Perspectives

Taken together, our results suggest that SQ plays a considerable role in ameliorating renal injury in I/R induced AKI through reducing apoptosis and enhancing autophagy, which are associated with regulating ERK/mTOR pathways. Future studies would be necessary to investigate more time points covering the entire I/R injury process, explore deeper mechanisms of SQ treatment and its active components, as well as the relationship of interlinked cell death pathways during I/R-AKI.

## Data Availability Statement

The raw data supporting the conclusions of this article will be made available by the authors, without undue reservation, to any qualified researcher.

## Ethics Statement

The animal study was reviewed and approved by the Institutional Animal Care and Use Committee of Guangdong Provincial Hospital of Chinese Medicine.

## Author Contributions

RT, WM, QH, and PX conceived and designed the study. RT, PW, LH, and ZYL performed the experiments. ZSL and AW assisted with some assays. RT, PW, and CL analyzed the data and wrote the manuscript. KB, WM, and PX contributed to the discussion and revision of the manuscript. All authors contributed to the article and approved the submitted version.

## Funding

This research was funded by Traditional Chinese Medicine Bureau of Guangdong Province (No. 20181133), National Natural Science Foundation of China (Nos. 81774216 and 81974565), the Specific Research Fund for TCM Science and Technology of Guangdong Provincial Hospital of Chinese Medicine (No. YN2016QJ12), Science and Technology Planning Project of Guangdong Province (Nos. 2018B030322012 and 2016A020226042), the Specific Fund of State Key Laboratory of Dampness Syndrome of Chinese Medicine (SZ2020ZZ04), Medical Scientific Research Foundation of Guangdong Province (A2020323), and the Key Project of High-level University Construction of Guangzhou University of Chinese Medicine (No. XK2019023).

## Conflict of Interest

The authors declare that the research was conducted in the absence of any commercial or financial relationships that could be construed as a potential conflict of interest.
