# Supplementary material for: Sanqi Oral Solution Ameliorates Renal Ischemia/Reperfusion Injury via Reducing Apoptosis and Enhancing Autophagy: Involvement of ERK/mTOR Pathways
Source: Front Pharmacol. 2020 Sep 16;11:537147. doi: 10.3389/fphar.2020.537147 (PMC7525120; doi:10.3389/fphar.2020.537147)
Supplement: Supplementary file 1 [file DataSheet_1.pdf]

Supplementary Table and Figure

Table S1 | Prescription of Sanqi oral solution (三芪口服液)

**Latin name**	**Botanical name**	**Chinese name**	**Concentration** (crude drug)
Radix Astragali	*Astragalusmembranaceus(Fisch.) ex Bunge*	Huang qi (黄芪)	0.333 g/mL
Rdix Notoginseng	*Panax notoginseng (Burk.) F. H. Chen*	Sanqi (三七)	0.056 g/mL

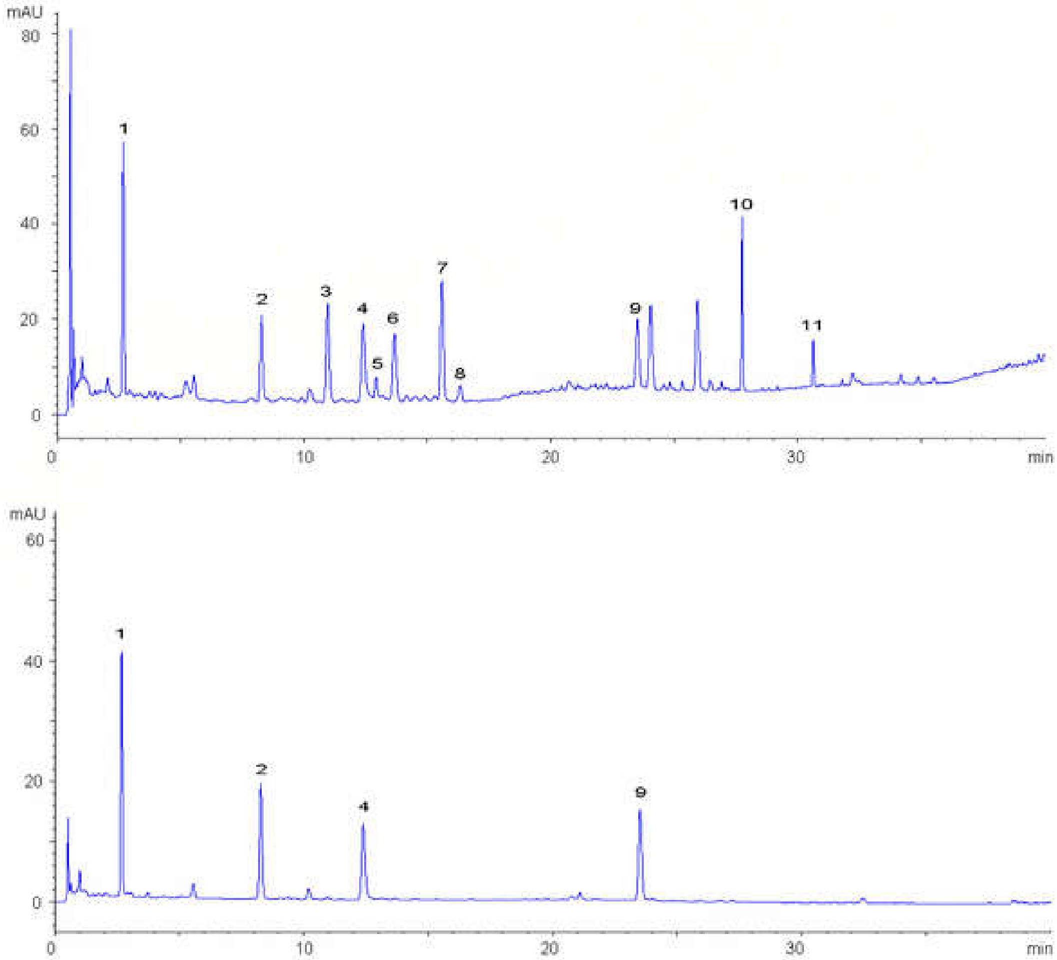

Figure S1 | UV chromatogram of SQ at 205 nm and 284 nm. 11 peaks were identified as: (1) caylcosin-7-O-β-D-glucopyranoside; (2) ononin; (3) 6aR, 11aR)-3-hydroxy-9,10-dimethoxypterocarpan-3-O-β-D- glucopyranoside; (4) calycosin; (5) notoginsenoside R1; (6) isomucronulatol-7-O-β-D-glucopyranoside; (7) ginsenoside Rg1; (8) ginsenoside Re; (9) formononetin; (10) ginsenoside Rb1; (11) ginsenoside Rd.
